# Democratic Ideals

**Published:** 1918-03-09

**Authors:** 


### NURSING AFFAIRS IN IRELAND.

### DEMOCRATIC IDEALS.

### WHERE THE GUINEA STAMP RULES IN NURSING.

IN these days of tumult the word Democracy looms large, and many pin their faith to the Latin proverb that the voice of the people is the voice of the gods. This is a good enough working definition, but it is not universally true, else the cry "Not this man, but Barabbas," would never have been heard from the multitude. Minorities are sometimes right. To the nursing profession it is important that its members should have a sound conception of the true meaning of the word which is freely used by camp leaders whose aims are fundamentally opposed. The underlying principle of true Democracy is the enthronement of merit, and, if this is observed, it matters little whether the function of selection is entrusted to an individual or to a crowd.

Miss Eden voices many noisy people now appealing to British nurses in the name of Democracy, and with great astuteness they have chosen this war-cry because it tickles the popular fancy. Of their interpretation of its meaning there is only one practical and final test—an analysis of the principles and practices of such Associations as they champion. Miss Eden and her friends have nailed their colours to the mast of the Irish Nurses' Association, and they do so to the extent of demanding its recognition as a condition to a State Registration Bill for nurses. This is not a trifling matter or a quibble. Contentious Bills during the war are barred by Parliament, and hence avowed obstructionists owe it to the nursing profession to demonstrate the justice of their attitude. The Irish Nurses' Association have more than once sent representatives to London to contend for their alleged democratic ideals, and have displayed such skill in faction fighting as would suggest an upbringing in the region of Donnybrook. This is the only achievement that I know of standing to their credit, and if there be aught else it is up to them now to publish it, lest it fall into oblivion.

Let us examine this ideal of Democracy. What are the conditions of membership, and what the system of government? In a democratic institution one would have thought a diploma of training and a satisfactory character reference sufficient to establish a claim to membership. This is not the case. The most exclusive social club in the kingdom does not possess a more rigid standard than that of the I.N.A. The printed rules contain not a word about training: not a word about character. It is simply provided that candidates for membership "shall be elected by the unanimous vote of the Executive Committee." One black bean kills. The friendless nurse can imagine with what trepidation and misgivings she would fill up a form of application to be submitted to such a directory. The next question is, Who comprise the Executive, and how do they arrive? The answer to this is that the Executive formed itself first, and as all the members are unanimously approved, the governing body carry on from year to year without any change save such as may be inevitable owing to death or removal to another locality. The democratic mind of the Association is eloquently and pithily set out in its first rule: "The affairs of the Association shall be in the hands of the President and Executive Committee"! The much approved members count, and have always counted, both according to the printed rules and in actual practice, for less than nothing.

But lest peradventure nurses should at any time succeed in obtaining admission and afterwards revolt against the tyranny of the Executive, this astute body make abundant provision for the preservation of their authority. Executive Committee members pay a subscription of a guinea a year, whilst what are described as "ordinary members" pay only half-a-crown. This is a democracy where "rank is but the guinea stamp" in very truth. And as a guinea is roundly the value of eight half-crowns, so accordingly the vote of one member of the Executive is a set-off against eight votes of "ordinary" members. Here is the text: "When a division takes place only one representative for each eight members other than guinea subscribers may vote." They do not put a tooth on their golden principles. At this point I turn automatically to the composition of the Executive, and I find in the Report which I have before me—that for the year ending St. Patrick's Day, 1916—that there are twenty-seven members, so that in order to carry any motion contrary to their wishes there must be a net majority of at least 217 "ordinary" members. The nurses would have to "down tools" and desert their hospitals in order to turn out the Government! The Executive, moreover, is composed mainly of matrons, who can always regulate the attendance of the rank and file by granting leave of absence from duty. Further comment is superfluous.

It is in support of this ideal that I challenge Miss Eden to enter a defence, and I promise to pay for my perfidy if I am adjudged to be in the wrong. It is an easy matter to camouflage readers of the *Manchester Guardian*, who know nothing of the facts, but hardly necessary to point out that Miss Eden's words fall on deaf ears in Ireland.

Miss Eden clamours against the laity, or persons other than fully-trained nurses, meddling with such matters as these. Is Miss Eden herself a fully-trained nurse? There are those who say that she is not. I have no hesitation in confessing that I belong to the lay abomination, and if Miss Eden is ditto I shall think no worse of her on this account. I have stated my case; it is for her to reply. IERNE.

